# Lactate and Lactylation in Pulmonary Hypertension: Comprehensive Landscape and Future Perspectives

**DOI:** 10.7150/ijms.125397

**Published:** 2026-06-04

**Authors:** Qing Dai, Yichao Cai, Xinyu Wang, Xia Li, Runxiu Zheng, Xianya Cao, Chao Zhang, Jun Xu, Jian Yi, Lan Song, Aiguo Dai

**Affiliations:** 1 School of Medicine, Hunan University of Chinese Medicine, Changsha, China.; 2 Hunan Provincial Key Laboratory of Vascular Biology and Translational Medicine, Changsha, China.; 3 School of Medicine, Ningxia Medical University, Ningxia, China.; 4 Department of Gerontology Medicine, The First Affiliated Hospital of Hunan University of Chinese Medicine, Changsha, China.; 5 Department of Respiratory Medicine, The First Hospital of Changsha, China.

**Keywords:** lactate/lactylation, pulmonary hypertension, PASMCs proliferation, immune inflammatory, pulmonary fibrosis, vasoconstriction

## Abstract

Pulmonary hypertension (PH) is a progressive pulmonary vascular disease with a poor prognosis and limited treatment options. Emerging evidence suggests that metabolic reprogramming plays a central role in driving PH pathogenesis. Among the key metabolic factors, lactate—the end product of glycolysis—has gained increasing recognition as a crucial regulator linking cellular metabolism to functional activity. Additionally, lactylation, a newly identified post-translational modification associated with lactate metabolism, has been shown to influence protein function and gene expression, further implicating its role in PH. While previous reviews have introduced these concepts, a systematic framework that directly integrates these molecular mechanisms into the core pathological hallmarks of the disease has been notably absent. In this review, we systematically examine the potential molecular mechanisms by which lactate and lactylation contribute to PH pathogenesis. Specifically, we systematically dissect the multifaceted roles of lactate and lactylation through the lens of five distinct pathological pillars of PH: (1) aberrant proliferation of pulmonary artery smooth muscle cells (PASMCs), (2) dysregulated immune-inflammatory responses, (3) progressive pulmonary fibrosis, (4) abnormal vasoconstriction, and (5) PASMC ferroptosis. By structuring our analysis around these core processes, we offer a novel, integrated perspective on how a single metabolic axis—lactate and lactylation—concertedly drives the complex pathophysiology of PH. This review aims to provide a comprehensive and forward-looking perspective that may guide future research into PH pathophysiology and treatment.

## Introduction

Pulmonary hypertension (PH) is a complex clinical syndrome characterized by progressive remodeling of the pulmonary vasculature and increased vascular resistance, ultimately leading to right ventricular hypertrophy, heart failure, and death. Epidemiological studies estimate that PH affects approximately 1% of the global population, posing a significant health burden across all age groups 1. Notably, its prevalence rises sharply in individuals aged ≥ 65 years, reaching nearly 10% 2. According to the World Symposium on Pulmonary Hypertension (WSPH), PH is clinically classified into five distinct groups: Pulmonary Arterial Hypertension (PAH, Group 1), PH due to left heart disease (Group 2), PH due to lung diseases and/or hypoxia (Group 3), PH due to pulmonary artery obstructions (Group 4), and PH with unclear and/or multifactorial mechanisms (Group 5). In this review, we use the term “PH” to refer to the broad clinical syndrome.

PH is associated with a poor prognosis. While the standardization of targeted therapies in China has improved the 3-year survival rate of idiopathic PH to 75.1%, this progress is largely limited to Group 1 PH. The most prevalent clinical subtypes—PH associated with left heart disease (Group 2) and lung diseases (Group 3)—still lack approved specific treatments 3. Therefore, a deeper understanding of the molecular mechanisms underlying PH, along with the identification of early diagnostic biomarkers and novel therapeutic targets, is essential for improving patient outcomes.

**Figure 1:**
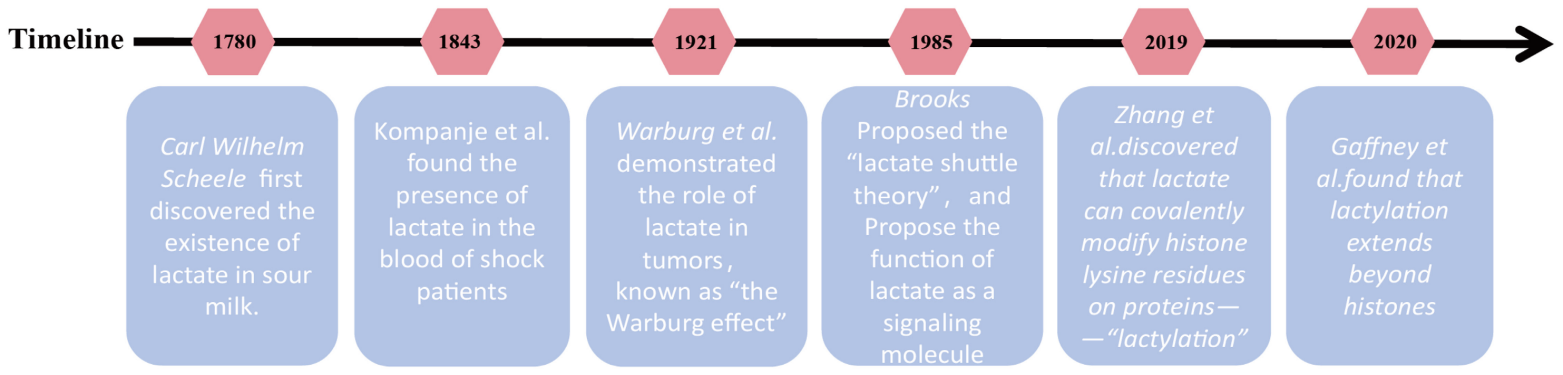
** History of the discovery and development of lactate and lactylation.** Representative milestone events leading to the discovery and development of lactate and lactylation are enumerated in the figure. Key milestones include: (1) 1780: Carl Wilhelm Scheele first discovered lactate in sour milk. (2) 1843: Johann Scherer detected lactate in human blood during shock, initially fueling its reputation as a harmful byproduct of hypoxia. (3) 1921: Otto Warburg described the "Warburg Effect," noting that tumor cells preferentially produce lactate even in the presence of oxygen. (4) 1986: George Brooks proposed the "Lactate Shuttle Theory," identifying lactate as a key energy substrate and signaling molecule between tissues. (5) 2019: A breakthrough study by Zhang *et al*. identified histone lysine lactylation (Kla) as a novel epigenetic modification, directly linking cellular metabolism to gene transcription. (6) 2020: Gaffney *et al*. discovered that lactylation also occurs on non-histone metabolic enzymes, exerting negative feedback on glycolytic flux.

Lactate was first isolated as an organic acid from sour milk by Carl Wilhelm Scheele. In 1843, Johann detected lactate in the blood of patients in shock, contributing to the early misconception that lactate is merely a "harmful metabolic waste product" generated under hypoxic conditions 4. However, this perception began to shift in 1921 when Otto Warburg demonstrated that tumor cells preferentially metabolize glucose into lactate via aerobic glycolysis, underscoring its pivotal role in tumor metabolism 5. By the 1970s, George Brooks challenged conventional wisdom by proving that lactate is not simply the end product of anaerobic metabolism but rather a key intermediate in glucose metabolism. His findings revealed that lactate can enter mitochondria and fuel oxidative processes, directly serving as an energy substrate for tissues such as neurons, cardiac muscle, and skeletal muscle 6. Beyond its metabolic role, lactate has also emerged as a critical signaling molecule, regulating processes such as immunity, inflammation, and apoptosis 7. A major breakthrough came in 2019 when Professor Zhang and colleagues discovered that lactate can covalently modify lysine residues on proteins, unveiling a novel epigenetic mechanism termed “lactylation” (Kla) 8. This discovery significantly broadened our understanding of lactate signaling, highlighting its ability to regulate gene expression and alter protein function. Moreover, it shed new light on the intricate interplay between metabolites and gene regulation, revealing an expanded role for lactate in disease progression. The key historical milestones in the discovery and research of lactate and lactylation are summarized in Figure 1.

PH is characterized by pathological pulmonary vascular remodeling and cells in the pulmonary vasculature are essential for maintaining vascular homeostasis, including pulmonary artery endothelial cells (PAECs), pulmonary artery smooth muscle cells (PASMCs), fibroblasts, and immune cells 9. Recent research has increasingly highlighted the crucial role of pulmonary vascular metabolic homeostasis, emphasizing that metabolic dysregulation is closely linked to the pathogenesis of PH. In particular, the metabolic reprogramming of pulmonary vascular cells has been shown to profoundly influence cellular behaviors and phenotypes, thereby driving PH progression 10, 11. Among the key metabolic factors involved, emerging evidence suggests that lactate plays a pivotal role in bridging metabolic reprogramming with PH development. Moreover, lactylation, a post-translational modification associated with lactate metabolism, has recently been recognized as a significant factor in the pathophysiology of PH, further underscoring the intricate metabolic mechanisms underlying the disease. While a recent review by Peng *et al*. 12 has provided a valuable overview of lactate metabolism and lactylation in PH, a systematic framework linking these molecular events to the core pathological processes of the disease is still lacking. Our review fills this gap by uniquely structuring the discussion around five key pillars of pulmonary vascular remodeling in PH: PASMC proliferation, immune-inflammatory responses, pulmonary fibrosis, abnormal vasoconstriction, and PASMC ferroptosis.

## Overview of Lactate

### Metabolism of Lactate

Under physiological conditions, glucose is metabolized to pyruvate via glycolysis, yielding a net gain of two adenosine triphosphate (ATP) and two nicotinamide adenine dinucleotide (NADH, reduced form) molecules. When oxygen is abundant, pyruvate typically enters the mitochondria for complete oxidation via the tricarboxylic acid (TCA) cycle. However, under hypoxic conditions or during metabolic reprogramming, pyruvate is preferentially diverted toward lactate generation. Biochemically, this reaction is catalyzed by lactate dehydrogenase (LDH), which reduces pyruvate to lactate coupled with the oxidation of NADH to oxidized nicotinamide adenine dinucleotide (NAD^+^). This regeneration of cytosolic NAD^+^ is a critical rate-sustaining step, as it replenishes the electron acceptor pool required for the glyceraldehyde-3-phosphate dehydrogenase (GAPDH) reaction, thereby maintaining continuous glycolytic flux and rapid ATP production 13. Beyond glycolysis, glutaminolysis serves as an additional source of lactate 14. Upon entering the cytoplasm, glutamine is first processed by glutaminase (GLS) and glutamate dehydrogenase (GLUD) to generate alpha-ketoglutarate (α-KG), which then enters the TCA cycle. Within the TCA cycle, glutamine-derived carbon is metabolized into oxaloacetate and subsequently malate. Malate then exits the mitochondria and is converted by cytosolic malic enzyme 1 (ME1) into nicotinamide adenine dinucleotide phosphate (NADPH) and pyruvate, with the latter serving as a precursor for lactate synthesis. This pathway allows glutamine to contribute carbon skeletons for lactate production, establishing it as a secondary source of lactate.

**Figure 2:**
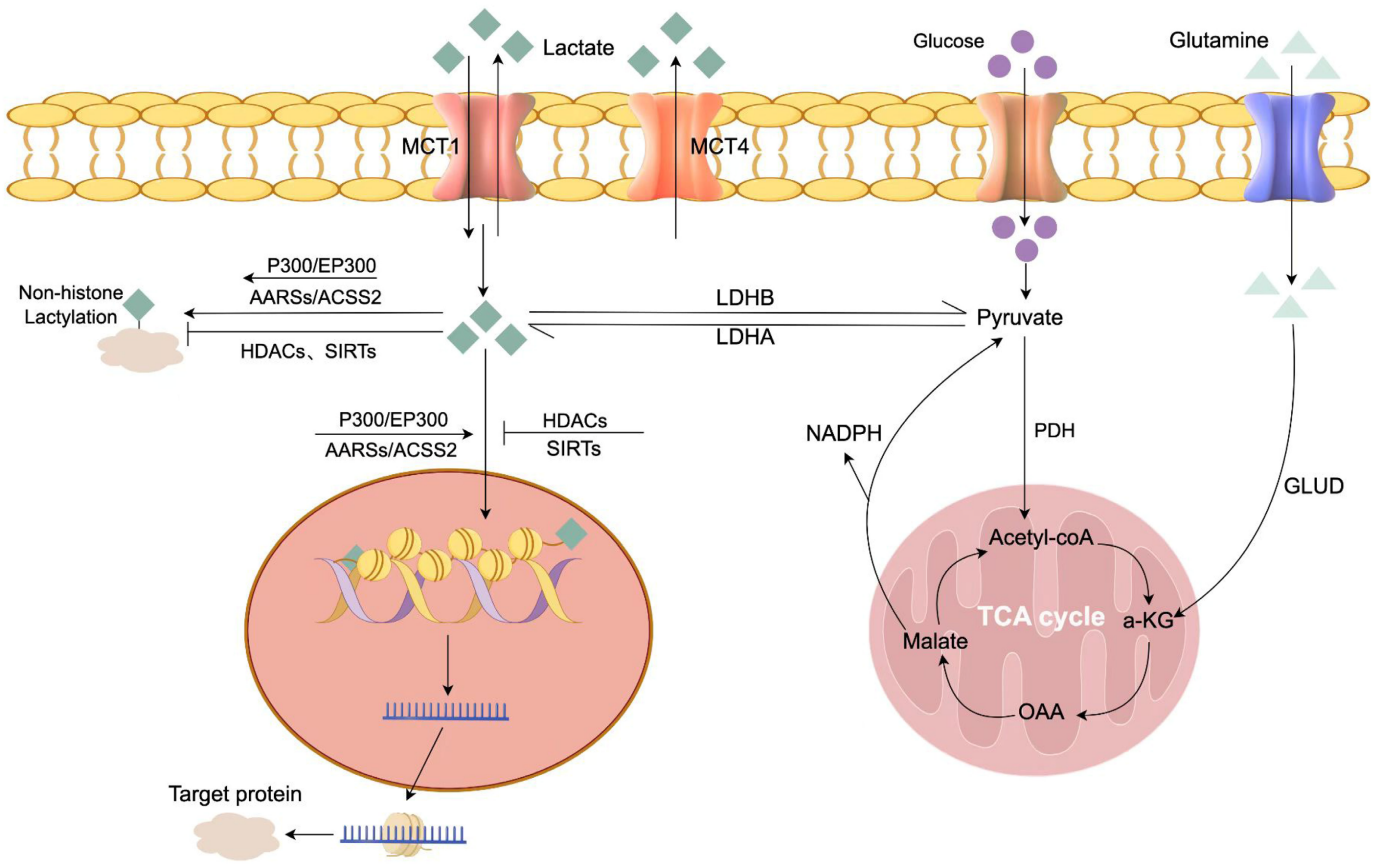
** Metabolism of lactate and lactylation.** This schematic provides a comprehensive overview of lactate homeostasis and its conversion into lactylation modifications. Intracellular lactate is primarily produced from glucose via glycolysis (catalyzed by LDH) or secondary sources like glutaminolysis, and it can be converted back to pyruvate or cleared via the PDH complex to enter the TCA cycle for oxidative phosphorylation. The transmembrane flux of lactate is regulated by specific transporters: MCT1 directs lactate transport according to the transmembrane lactate gradient, while MCT4 is responsible for lactate efflux. These metabolic dynamics are coupled with the regulation of protein lactylation, where p300/EP300, ACSS2, and AARS1/2 function as lactoyltransferases that catalyze the transfer of lactyl groups to lysine residues on target proteins, whereas HDACs and sirtuins act as delactylases that catalyze the removal of these modifications. Abbreviations used in the figure: AARSs: Aminoacyl-tRNA synthetases; MCT1/4: Monocarboxylate transporter 1/4; LDHA/B: Lactate dehydrogenase A/B; PDH: Pyruvate dehydrogenase; GLUD: Glutamate dehydrogenase;ɑ-KG: ɑ-ketoglutarate; OAA: Oxaloacetate; NADPH: Nicotinamide adenine dinucleotide phosphate; AARSs: Alanyl-tRNA synthetases (specifically AARS1 and AARS2); ACSS2: Acetyl-CoA synthetase 2; P300/EP300: E1A-binding protein p300; HDACs: Histone deacetylases;SIRTs: Sirtuins.

Excess lactate accumulation is harmful, with elevated serum levels potentially causing lactic acidosis 15. Therefore, lactate must be rapidly cleared from tissues and circulation through metabolic processes. Pyruvate dehydrogenase (PDH), the rate-limiting enzyme catalyzing pyruvate's oxidative decarboxylation to acetyl-CoA, is critical for lactate's irreversible clearance. Pyruvate from diverse metabolic sources is converted by PDH into acetyl-CoA, which enters the TCA cycle for thorough oxidation 16. The detailed metabolic pathways of lactate production, transport, and the enzymatic regulation of lactylation are illustrated in Figure 2.

### Regulation of Lactate

The balance between anaerobic and aerobic lactate oxidation fluxes critically determines systemic lactate levels. Thus, factors affecting glycolysis or PDH flux can alter lactate levels.

### PDH

PDH is pivotal for lactate clearance. The E1 alpha subunit (PDHA1) is an essential component of PDH complex. PDHA1 expression is critical for the functionality of the TCA cycle and mitochondrial oxidative phosphorylation 17. Loss of PDHA1 disrupts PDH enzymatic activity, halting pyruvate conversion to acetyl-CoA. This disruption impairs the TCA cycle and disturbs both glycolytic and PDH fluxes 18. PDH activity is tightly regulated by phosphorylation and dephosphorylation. Pyruvate dehydrogenase kinase (PDK) inhibits PDH activity by phosphorylating PDHA1 19, whereas pyruvate dehydrogenase phosphatase (PDP) restores PDH function by dephosphorylating PDHA1^20^. Additionally, increased PDHA1 acetylation further suppresses PDH activity, adding another layer of regulatory control to this key metabolic enzyme 21.

### LDH

LDH is a tetrameric enzyme that mediates the interconversion of pyruvate and lactate, and is composed of lactate dehydrogenase A (LDHA) and B (LDHB) subunits 22. Subunit A, encoded by *LDHA*, is localized in the cytoplasm, mitochondria, and organelles, primarily catalyzing the conversion of pyruvate to lactate. Subunit B, encoded by *LDHB*, is predominantly localized in mitochondria, catalyzing the conversion of lactate to pyruvate 23. Studies have shown that LDHA is upregulated in glycolytically active cells, facilitating lactate production 24, whereas high LDHB expression suppresses lactate production 25, 26.

### MCTs

Monocarboxylate transporters (MCTs) are the primary transmembrane proteins associated with lactate transport and belong to the solute carrier family 16 (SLC16) 27. Under physiological conditions, the coordinated action of MCT1-4 facilitates lactate shuttling between glycolytic and oxidative cells, thereby maintaining lactate homeostasis in tissues. In normal tissues, the high-affinity MCT1 directs lactate transport according to the transmembrane lactate gradient 28. MCT4 plays a crucial role in lactate efflux, lowering intracellular lactate levels by exporting lactate out of the cells 29.

## Overview of Lactylation

### Discovery of Lactylation

In 2019, lactylation was proposed as a novel histone modification. Zhang *et al* 8. first identified a 72.021 Da mass shift on histone lysine residues using mass spectrometry. They further confirmed the widespread presence of histone lactylation through stable isotope labeling, along with a series of *in vitro* and *in vivo* experiments. Additionally, their study revealed the presence of lactyl-CoA, suggesting that lactylation is an enzymatically regulated post-translational modification, with lactyl-CoA serving as its substrate. Based on these findings, they proposed that histone lactylation represents a new epigenetic mechanism for regulating gene transcription. In 2020, Gaffney *et al*. 30 later found that lactylation extends beyond histones, influencing a broad range of metabolic enzymes. Moreover, lactylation of these enzymes exerts negative feedback on the glycolytic pathway. Notably, the lactylation of these enzymes was found to exert a negative feedback effect on the glycolytic pathway, further underscoring its regulatory significance in cellular metabolism. (Figure 1).

### Modes of Lactylation

Lactylation can be classified into L-lactylation and D-lactylation, based on the two lactate stereoisomers. L-lactate is the primary glycolytic product, whereas D-lactate is a byproduct of the metabolic pathway 31. L-lactylation utilizes L-lactate as its precursor. Biologically, this process requires the activation of lactate into Lactyl-CoA, a high-energy thioester donor. Specific acyltransferases then catalyze the transfer of the lactyl moiety from Lactyl-CoA to the epsilon-amino group of lysine residues, forming a stable amide bond; this mechanism represents the canonical “enzymatic lysine lactylation” 8. In contrast, D-lactylation is derived from methylglyoxal (MGO). During glycolysis, glucose is converted into glyceraldehyde 3-phosphate (GAP) and dihydroxyacetone phosphate (DHAP), from which a portion of GAP and DHAP is non-enzymatically converted into the glycolytic byproduct MGO. MGO is then converted into lactoylglutathione (LGSH) by glyoxalase 1 (GLO1) with the assistance of glutathione 32, 33. LGSH derived from MGO is subsequently hydrolyzed by glyoxalase 2 (GLO2) to produce D-lactate and glutathione 33. However, when LGSH accumulates, the reactive lactyl group within its thioester bond can undergo a direct nucleophilic attack by lysine residues. This results in the transfer of the lactyl group without enzymatic assistance, a process defined as D-lactylation or “non-enzymatic lactylation” 30. It is currently believed that both L-lactylation and D-lactylation pathways can coexist in the same cell. Furthermore, L-lactate can inhibit lactoylglutathione degradation and elevate its levels, thereby promoting D-lactylation.

### Regulatory Mechanisms of Lactylation

Lactylation levels are primarily regulated by three core processes: lactate production, lactate transport, and the enzymatic balance between lactyltransferases and delactylases (lactate-removing enzymes).

### Lactate Production

Lactylation levels increase in a dose-dependent manner with rising lactate concentrations. Histone lactylation was initially considered a post-translational modification derived from endogenous lactate. However, later studies have shown that both exogenous sodium lactate and elevated endogenous lactate levels promote lactylation, with endogenous lactate having a more significant effect 8, 34. Therefore, lactate production and transport are key factors influencing lactylation. Since lactate is the end product of glycolysis, its production is primarily determined by the balance between glycolysis and mitochondrial oxidative phosphorylation. Consequently, changes in the activity of key enzymes in the glycolytic pathway significantly affect lactate production and, thus, lactylation levels. Studies have shown that during M1 macrophage polarization, lactate concentrations and overall lactylation levels are significantly reduced in LDHA knockout macrophages 8. Lactate production depends on the balance between glycolysis and the TCA cycle, with enhanced glycolytic activity typically leading to increased lactylation levels. The mitochondrial respiration inhibitor, rotenone can suppress the TCA cycle, increasing lactate accumulation and elevating lactylation levels 35, 36. In contrast, the non-metabolizable glucose analogue 2-deoxy-D-glucose (2-DG) inhibits glycolysis and significantly reduces lactylation levels 37, 38.

### Lactate Transport

Lactate transport is another critical factor influencing lactylation. As previously mentioned, lactate exists as two stereoisomers, L-lactate and D-lactate, both of which are transported between intracellular and extracellular environments by the MCTs family 39. MCTs play a key role in the uptake of extracellular lactate and in the lactylation process. L-lactate is converted into acetyl-CoA by acetyltransferases and participates in lactylation. The transport of L-lactate is mainly mediated by MCT1 and MCT2, which facilitate its influx from the extracellular space, while MCT4 promotes its efflux, regulated by the lactate concentration gradient 40-42. In the tumor microenvironment, hypoxic cancer cells produce lactate through LDHA and release it into the extracellular space via MCT4. Subsequently, normoxic cells take up lactate via MCT1 and convert it to pyruvate through LDHB, generating ATP 43. In sepsis, macrophages uptake extracellular lactate through MCTs, mediating the lactylation of high mobility group box 1 (HMGB1). Monocarboxylate transporter inhibitors can suppress MCTs activity, reducing macrophage uptake of extracellular lactate and decreasing HMGB1 lactylation levels 44. For cells with low endogenous lactate production, MCTs play a more significant role in lactylation.

### Lactyltransferases and Lactate-Removing Enzymes

In enzymatic pathways, lactate is believed to be converted into lactoyl-CoA via lactylation catalyzed by CoA-transferase 8. Regulatory enzymes that transfer lactoyl groups likely play a key role in regulating the extent of lactylation. Multiple enzymes involved in converting lactate to lactoyl-CoA have been identified in microbes, such as YdiF in *Escherichia coli* and ME-PCT in *Megasphaera elsdenii*^32^, ^45^. Recent studies show that alanyl-tRNA synthetases 1 (AARS1) and 2 (AARS2) are bona fide lactoyltransferases, functioning as L-lactate sensors by binding intracellular L-lactate and directly transferring the lactoyl group to lysine residues, thereby inducing lactylation^46^-^48^. Notably, acetyl-CoA synthetase 2 (ACSS2) has been identified as a lactoyltransferase that mediates lactylation and promotes tumor immune evasion 49. Additionally, E1A-binding protein P300 (EP300) and CREB-binding protein (CBP) act as lactoyltransferases, catalyzing the transfer of lactoyl groups to lysine residues 8, 44. Studies indicate that *EP300* knockout in human colorectal cancer HCT116 cells and HEK293T cells reduces global lactylation and H3K18la modification levels 8. Conversely, Varner *et al*. 50 reported that histone deacetylases (HDACs) act as “erasers” of lactylation, catalyzing the removal of ε-N-lactoyl modifications. Moreno-Yruela *et al*. 51 further demonstrated that HDAC1-3 efficiently remove lactylation, while class III histone deacetylases, such as sirtuin 1-3 (SIRT 1-3), also act as “erasers” of lactylation.

## Lactate and Lactylation in PH

Under physiological conditions, cellular energy metabolism primarily relies on oxidative phosphorylation. However, in PH, despite adequate oxygen availability, PAECs and PASMCs exhibit a metabolic shift characterized by enhanced glycolysis and diminished oxidative phosphorylation—a phenomenon termed the “Warburg effect”. This metabolic reprogramming results in elevated lactate production, contributing to disease progression.

### Lactate Levels in PH

#### Lactate in PH Serum

Multiple studies have demonstrated that serum lactate levels are significantly higher in PH patients compared to age-matched healthy controls 52-54. In elderly patients with PH associated with chronic obstructive pulmonary disease (COPD), arterial blood lactate levels show a positive correlation with pulmonary arterial systolic pressure 55, suggesting that serum lactate levels increase with PH severity. Beyond its correlation with disease progression, circulating lactate serves as a critical prognostic marker for PH. Patients with elevated lactate levels exhibit higher mortality rates and an increased risk of adverse cardiovascular events. Using a multi-omics approach, Anna Hemnes identified plasma lactate as a hallmark feature of right ventricular failure in PAH patients 52. Similarly, recent work by Deng *et al*. established hyperlactatemia as an independent predictor of mortality in connective tissue disease-associated PAH patients complicated with right heart failure 56. Findings from animal models further support these observations, demonstrating that serum lactate levels are consistently elevated in monocrotaline-induced PH 57. Collectively, these studies highlight the potential of blood lactate as a reliable biomarker for assessing PH severity and progression.

#### Lactate in PH Tissues

Metabolomic analyses conducted by Jose *et al*. 58 on lung and right ventricular tissues from hypoxia-induced PH mice, as well as hypoxia combined with semaxanib (SU5416)-induced PH mice, revealed elevated lactate levels compared to age-matched wild-type controls. Notably, lactate concentrations in lung tissue correlated closely with key histological features of PH, such as the medial wall thickness index. The increased lactate production observed in PH models is primarily driven by the enhanced expression and activity of LDHA. LDHA has been identified as a key mediator of lactate generation in PH animal models 59, 60. Furthermore, lactate accumulation in these models is LDHA-dependent, as genetic deletion or pharmacological inhibition of LDHA has been shown to mitigate vascular remodeling and right ventricular dysfunction in multiple PH models, including SU5416/hypoxia-induced mouse models, monocrotaline-induced rat models, and chronic hypoxia-induced mouse models. These findings underscore the potential of targeting lactate production as a therapeutic strategy to alleviate PH progression.

#### Lactate in PH Cells

Daniel *et al*. 61 utilized 13C-labeled isotope metabolomics to analyze PASMCs and PAECs isolated from PH patients. Their findings revealed increased ¹³C-labeled lactate production in PASMCs and PAECs, indicating a metabolic shift from oxidative phosphorylation to anaerobic glycolysis in PH. This metabolic reprogramming has been further corroborated by studies from Caruso and Smolders 62, 63. Similarly, multiple studies in animal models of PH have confirmed glycolytic reprogramming in PASMCs and PAECs, characterized by lactate accumulation 64-67. These findings underscore the conserved nature of metabolic dysregulation across different PH subtypes and models, highlighting glycolytic reprogramming as a fundamental feature of PH pathophysiology.

### Lactylation in PH

Hypoxia, a key pathogenic mechanism underlying PH, serves as a fundamental driver of its onset and progression 68, 69. Studies have shown that hypoxia induces a glycolytic shift, leading to increased lactate production, which in turn acts as a precursor for lactylation^8^.

Emerging studies revealed the dual role of lactylation in PH in regulating both chromatin dynamics and non-histone protein functions. Regarding histone lactylation, hypoxia stimulates broad hyper-lactylation, specifically at H3K18 and H4K5 residues, in PASMCs and lung tissues of hypoxic PH rats. These modifications function as epigenetic activators 67. Chen A *et al*. 70 recently provided causal evidence linking this metabolic-epigenetic axis to vascular remodeling. They identified that H3K18la is specifically enriched at the promoters of placental growth factor (*Pgf*) and *Ythdf2*, directly driving their transcription to fuel aberrant PASMC proliferation. Furthermore, in the specific context of obstructive sleep apnea (OSA)-associated PH, Yang *et al*. 71 demonstrated that hypoxia-induced lactate accumulation triggers lactylation at the phosphodiesterase 4B(*PDE4B*) promoter. This epigenetic event activates the downstream FUS/AGT signaling axis, thereby promoting vascular remodeling.

Beyond histones, current research highlights the critical impact of non-histone protein lactylation. Li *et al*. 72 uncovered a novel regulatory axis where the acetyltransferase EP300 mediates the lactylation of the RNA-binding protein FUS. This modification triggers liquid-liquid phase separation (LLPS), which suppresses the transcription of the antioxidant gene SLC7A11, thereby inducing ferroptosis-mediated vascular injury. Collectively, these findings underscore that lactylation orchestrates PH progression through a multi-layered network, coupling metabolic stress to epigenetic remodeling and protein functional changes. A comprehensive summary of lactate levels and lactylation changes observed in PH patients, animal models, and cellular models is provided in Table 1.

### Regulation of Lactate and Lactylation in PH

#### Regulation of Lactate in PH

Lactate production and metabolism are disrupted in PH, characterized by altered activities of LDH and PDH. Clinical studies report elevated serum LDH levels in PAH patients, significantly contributing to increased serum lactate concentrations 52, 56. Additionally, PDH inactivation is a hallmark of PH and is mediated by the enhanced activity of PDK. PDK phosphorylates the PDHA1, thereby inhibiting PDH activity and redirecting pyruvate away from the TCA cycle toward lactate production 73, 74. Notably, the PDK inhibitor dichloroacetate (DCA) restores PDH activity and reduces lactate production in PASMCs 73. Beyond enzymatic dysregulation, aberrant lactate transport also plays a critical role in PH. Dysregulation of MCT4 has been observed, with increased MCT4 expression detected in hypoxia-induced PH models and hypoxia-treated PAECs 75. Similarly, PDGF-treated PASMCs exhibit upregulated MCT4 protein levels, facilitating lactate efflux 76. Importantly, MCT4 inhibition reduces lactate export while increasing intracellular lactate accumulation in PASMCs, highlighting MCT4 as a potential therapeutic target in PH 77.

#### Regulation of Lactylation in PH

In PH, lactylation is primarily regulated by lactate levels and EP300. For instance, treatment with oxamate, an LDH inhibitor, reduces elevated H3K18la and H4K5la in pulmonary artery homogenates from hypoxic PH rats 67. Similarly, silencing *PDK1/2*, key PDH inhibitory kinases, lowers these lactylation levels—an effect that is reversible upon exogenous L-lactate incubation 67. Mechanistically, this process is driven by mitochondrial reactive oxygen species (mROS); clearance of mROS with MitoQ mitigates hypoxia-induced increases in H3K18la and H4K5la. This protection is likely due to mROS-mediated activation of HIF-1ɑ, which enhances LDHA expression while suppressing PDH activity, thereby promoting lactate accumulation over mitochondrial oxidative metabolism 67. This substrate-dependent regulation is further corroborated by recent studies, Yang *et al*. 71 showed that hypoxia-induced lactate accumulation was shown to specifically fuel lactylation at the *PDE4B* promoter, confirming that metabolic flux strictly controls the epigenetic landscape. Beyond lactate regulation, EP300 plays a pivotal role specifically in PH-related lactylation. Li *et al*. 72 demonstrated that EP300 expression is significantly upregulated in hypoxic pulmonary vascular cells. Functionally, EP300 was shown to directly catalyze the lactylation of key regulatory proteins (such as the RNA-binding protein FUS). Importantly, silencing EP300 or inhibiting its activity with C646 (P300/CBP inhibitor) effectively abolished these pathological lactylation events and reversed vascular remodeling traits in PH.

### Potential Molecular Mechanisms of Lactate and Lactylation in PH

**Figure 3:**
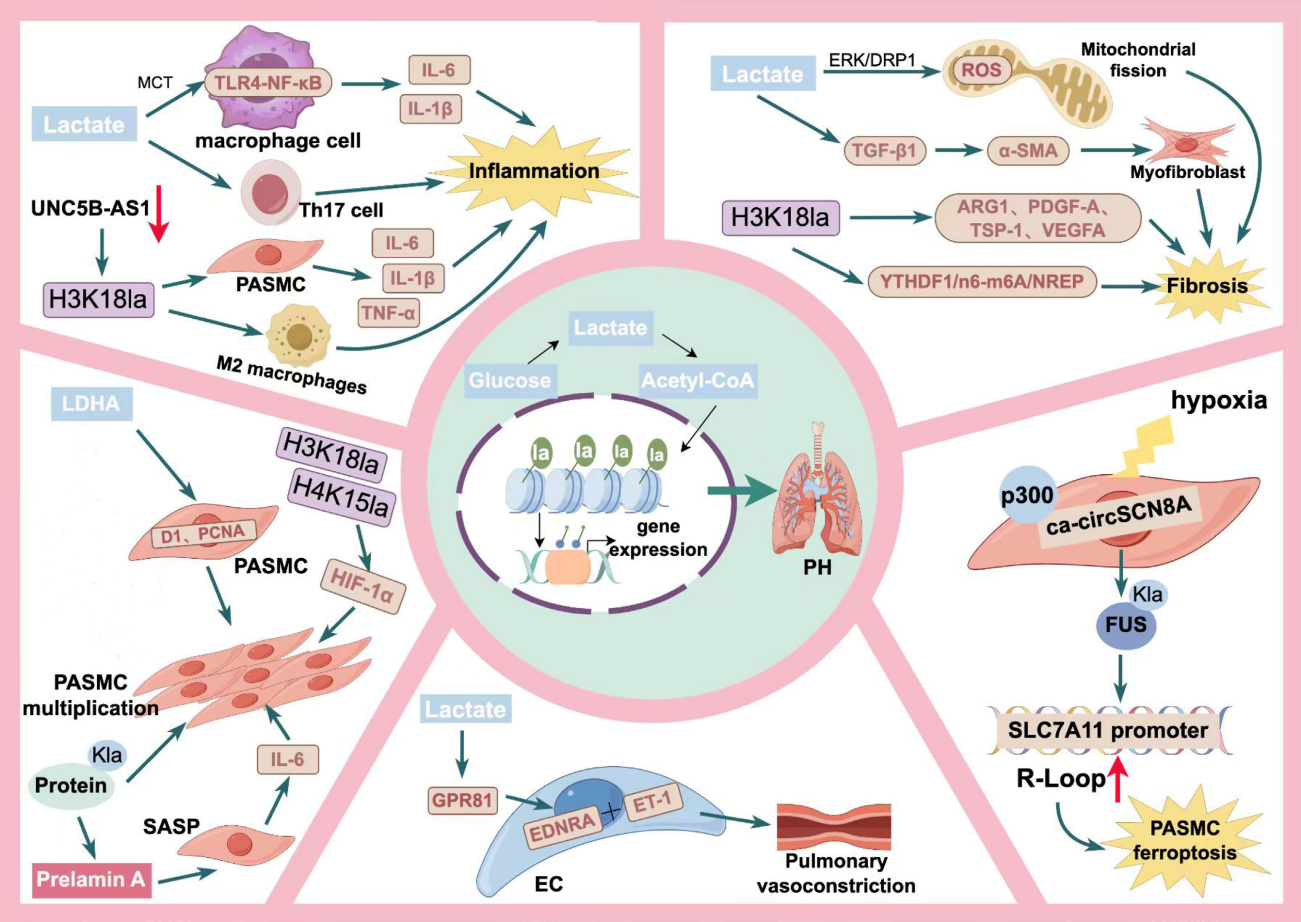
** Potential molecular mechanisms of lactate/lactylation in PH.** This conceptual model illustrates how the lactate-lactylation axis acts as a central metabolic hub driving PH pathogenesis through five distinct pathological pillars. In PASMC Proliferation, lactate stimulates proliferation in a concentration-dependent manner by elevating D1 and PCNA levels. H3K18la, H4K15la and protein Kla directly fuel this hyper-proliferative phenotype. Additionally, histone lactylation upregulates Prelamin A to induce PASMC senescence, which subsequently triggers a SASP and the release of IL-6 to stimulate neighboring cell proliferation via paracrine signaling. For the immune-inflammatory response, lactate facilitates TLR4-NF-κB signaling and activates Th17 cells. Concurrently, H3K18la triggers the release of pro-inflammatory cytokines (including IL-6, IL-1β, and TNF-α) from PASMCs and promotes the activation of M2 macrophages. In pulmonary fibrosis, lactate promotes myofibroblast differentiation through the ERK/DRP1-ROS and TGF-β1 pathways. Macrophage H3K18la further drives the expression of pro-fibrotic factors (ARG1, PDGF-A, TSP-1, VEGFA) and mediates progression via the YTHDF1/N6-methyladenosine (m6A)/NREP pathway. Regarding abnormal vasoconstriction, lactate activates GPR81 on endothelial cells to stimulate ET-1 release, which acts on EDNRA receptors to cause pulmonary vasoconstriction. Finally, in PASMC ferroptosis, hypoxia-induced *ca-circSCN8A* recruits p300 to catalyze FUS Kla, leading to R-loop formation and SLC7A11 promoter suppression, ultimately triggering iron-dependent cell death. Abbreviations used in the figure:Acetyl-CoA: acetyl-coenzyme A; ARG1: arginase 1; ɑ-SMA: ɑ-smooth muscle actin; D1: cyclin D1; DRP1: dynamin-related protein 1; EC: endothelial cell; EDNRA: endothelin receptor type A; ERK: extracellular regulated protein kinases; ET-1: endothelin-1; GPR81: G-protein coupled receptor; H3K18la: H3K18 lactylation; H4K15la: H4K15 lactylation; HIF-1ɑ: hypoxia inducible factor-1ɑ; IL-1β: interleukin-1β; IL-6: interleukin-6; Kla: lactylation; LDHA: lactate dehydrogenase A; MCT: monocarboxylate transporter; PASMC: pulmonary artery smooth muscle cells; PCNA: proliferating cell Nuclear antigen; PDGF-A: platelet derived growth factor subunit A; PH: pulmonary hypertension; ROS: reactive oxygen species; TGF-β1: transforming growth factor-β1; Th17 cell: T helper cell 17; TSP-1: thrombospondin-1; VEGFA: vascular endothelial growth factor A.

The pathogenesis of PH is complex, involving multiple cell types and diverse molecular pathways. Lactate, a key metabolic product of glycolytic reprogramming and a precursor of lactylation, establishes a critical link between lactylation modifications and glycolytic metabolism. Together with lactylation, lactate contributes to the pathophysiological processes underlying PH. As depicted in Figure 3, we propose that the lactate-lactylation axis drives PH pathogenesis through five key mechanisms: PASMC proliferation, immune-inflammatory responses, pulmonary fibrosis, abnormal vasoconstriction, and PASMC ferroptosis.

### Lactate/Lactylation and PASMC Proliferation

Abnormal proliferation of PASMCs represents a pivotal feature of pulmonary vascular remodeling, a key pathological process in PH 78. Emerging evidence reveals that intracellular lactate modulates PASMC function by inhibiting SENP1, stabilizing APC4 SUMOylation, and promoting UBE2C-APC/C interaction, a key complex for degrading mitotic cyclins. This process accelerates cyclin degradation, facilitating the mitosis-to-G1 phase transition. Sustained lactate accumulation, however, may drive aberrant APC/C remodeling, fostering uncontrolled cell proliferation and division. This mechanism appears to be a general paradigm through which intracellular lactate regulates cell cycle progression and proliferation in proliferative diseases 79.

Wu *et al*. 60 supported this hypothesis by showing that lactate stimulates PASMC proliferation in a concentration-dependent manner. Lactate treatment elevated cyclin D1 and PCNA protein levels in mouse PASMCs (mPASMCs). Moreover, lactate enhanced mPASMC migration via activation of the Akt signaling pathway. Intriguingly, knockdown of *LDHA*, an essential enzyme in lactate production, suppressed hypoxia-induced PASMC proliferation and migration. Importantly, LDHA depletion lowered lactate levels in the plasma and lung tissues across three distinct PH models—SU5416/hypoxia mouse, monocrotaline-induced rat, and chronic hypoxia-induced mouse models—thereby reversing pulmonary vascular remodeling. Consistently, Dong *et al* 80 also confirmed that lactate accumulation directly promotes PASMC proliferation. Their study demonstrated that this metabolic shift supports the hyper-proliferative and apoptosis-resistant phenotype by inhibiting AMPK and activating the AKT/mTOR signaling axis. These findings highlight the pivotal role of lactate in driving PASMC proliferation and vascular remodeling, contributing to PH progression.

Lactylation has also been implicated in promoting PASMC proliferation and migration, contributing to the pathological processes of PH. Elevated levels of H3K18 and H4K5 lactylation were observed in hypoxia-induced PASMCs and in PH rat models, as previously mentioned 67. Crucially, studies in 2025 have significantly expanded this mechanistic landscape. First, Chen *et al*. 70 provided direct causal evidence linking the “Warburg effect” to epigenetic remodeling. They demonstrated that hypoxia-induced global lactylation is a primary driver of PASMC proliferation, and inhibiting glycolysis with oxamate effectively abolished this phenotype, positioning the metabolic-epigenetic axis as a reversible therapeutic target. Similarly, in the specific context of OSA-associated PH, Yang *et al*. 71 identified that hypoxia triggers lactylation at the *PDE4B* promoter. This epigenetic event activates the downstream FUS/AGT signaling axis, thereby fueling vascular remodeling and highlighting a novel therapeutic target for OSA-induced PH. Furthermore, emerging evidence highlights a complex link between lactylation, cellular senescence, and vascular remodeling. Zhang *et al*. 81 revealed that lactylation directly upregulates Prelamin A, inducing PASMC senescence. Paradoxically, these senescent cells acquire a senescence-associated secretory phenotype (SASP) and release IL-6, which functions in a paracrine manner to stimulate the proliferation of neighboring PASMCs. This finding suggests that lactylation drives vascular expansion not only through direct mitotic entry but also via senescence-mediated intercellular signaling. Collectively, this evidence delineates a multi-layered regulatory network through which lactylation orchestrates pathological vascular remodeling.

### Lactate/Lactylation and Immune-Inflammatory Responses in PH

Immune-inflammatory responses have recently become a central focus in understanding PH pathogenesis 82, 83. Investigations have identified significant lymphocyte aggregation in the lungs of PAH patients, with circulating inflammatory cytokine levels strongly linked to poor clinical outcomes 84, 85. Moreover, in monocrotaline induced PH mice, immune cell-driven perivascular inflammatory infiltration often precedes structural pulmonary vascular remodeling 86, highlighting the pivotal role of immune-inflammatory mechanisms in PH. While lactate can establish an immunosuppressive microenvironment, excessive lactate exposure exacerbates inflammation-induced cellular damage 87.

Elevated Toll-like receptor 4 (TLR4) expression in PAECs, PASMCs, and immune cells, along with the activation of the TLR4-NF-κB signaling pathway, is a widespread pathological feature of PH. This has been consistently demonstrated across diverse disease contexts, including clinical samples from patients with COPD-associated PH, *in vivo* monocrotaline-induced animal models, and *in vitro* PDGF-stimulated PASMCs 88-90. Studies have demonstrated that lactate, via MCTs, facilitates TLR4 signaling in macrophages, leading to NF-κB-mediated gene transcription 91, 92. This process enhances the secretion and release of inflammatory cytokines, thereby exacerbating the inflammatory response. In addition, lactate has been shown to promote CD4⁺ T cell polarization into Th17 cells 93, 94. Notably, accumulating evidence suggests specific PH subtypes, such as systemic sclerosis (SSc)-associated and hypoxia-induced PH, are critically driven by Th17 cell-mediated immune responses 95, 96. Accordingly, targeting Th17 cells has been shown to ameliorate the disease in mouse models of pulmonary fibrosis-induced PH 97. These findings indicate that lactate may contribute to PH progression by activating the TLR4-NF-κB axis and driving Th17 cell-mediated immune responses, highlighting its potential role in PH pathogenesis.

Similarly, lactylation plays a critical role in the pathological processes of immune-inflammatory responses. Elevated M2 macrophage polarization markers have been observed in the lung tissues of patients with idiopathic PH 98-100, and an imbalance in the M1/M2 macrophage ratio is considered a key mechanism driving PH development. Recent studies indicate that lactylation profoundly influences M1/M2 macrophage polarization 101 and plays a critical role in regulating macrophage activity 102. While lactylation enhances M1 polarization in the early stages of inflammation, sustained lactate accumulation during M1 polarization induces histone H3 lactylation at lysine 18. This modification promotes the expression of M2-associated genes 8 and activates M2 macrophages 101, 103. Although some of these mechanisms were initially identified in tumor or fibrosis models 102, their relevance to PH is profound: the resulting accumulation of M2 macrophages leads to the secretion of pro-fibrotic and pro-proliferative factors. This microenvironmental shift facilitates pulmonary vascular remodeling, potentially accelerating PH progression. In addition, lactylation of pyruvate kinase M2 (PKM2) has been shown to regulate macrophage metabolic reprogramming, driving the transition from pro-inflammatory M1 polarization to pro-fibrotic M2 polarization 104. Thus, a hypothesis has emerged suggesting that lactylation promotes macrophage M2 polarization, which in turn stimulates PASMC proliferation and migration, ultimately contributing to PH progression. Notably, emerging evidence has revealed that lactylation-mediated inflammation extends to vascular resident cells, regulated by non-coding RNAs. A recent study identified the long non-coding RNA *UNC5B-AS1* as a critical regulator of lactylation in PASMCs. Mechanistically, the downregulation of *UNC5B-AS1* in hypoxic PH leads to the aberrant accumulation of H3K18la at the promoter regions of pro-inflammatory cytokines. This specific epigenetic modification activates the transcription of IL-1β, IL-6, and TNF-ɑ, thereby driving PASMCs toward a pro-inflammatory phenotype. This finding underscores that lactylation serves as a direct epigenetic switch governing the inflammatory transition of pulmonary vascular cells 105.

### Lactate/Lactylation and Pulmonary Fibrosis in PH

Fibrosis, characterized by excessive accumulation of extracellular matrix (ECM) components, is a common pathological feature in PH. Clinically, pulmonary hypertension is frequently observed secondary to pulmonary fibrosis, where it significantly worsens prognosis 106. Similarly, pulmonary fibrosis has been observed in the lung tissues of PH mouse models, and alleviating pulmonary fibrosis significantly improves PH outcomes 107.

Emerging evidence suggests that myofibroblasts in fibrotic lungs undergo pronounced cellular acidification, implicating lactate in the progression of pulmonary fibrosis 108, 109. Studies suggest that lactate promotes pulmonary fibrosis through the ERK/DRP1 signaling pathway, which enhances mitochondrial fission-derived reactive oxygen species (ROS) production 110. Furthermore, Robert *et al*. demonstrated that lactate supplementation in fibroblast culture media activates TGF-β1, a pivotal mediator of fibrosis, thereby promoting the differentiation of fibroblasts into myofibroblasts 111. Additionally, pharmacological inhibition of lactate production suppressed TGF-β1-induced α-SMA protein synthesis in pulmonary fibroblasts 112. Notably, blocking lactate production also prevented the progression of experimental pulmonary fibrosis in mouse models 113, 114. These findings underscore lactate as a crucial regulator of pulmonary fibrosis and propose lactate metabolism as a promising therapeutic target in PH-associated fibrosis.

Lactylation is intimately linked to cellular fibrosis. In fibrotic lungs, lactate secreted by myofibroblasts induces H3K18 lactylation in macrophages, driving the expression of pro-fibrotic factors, including ARG1, PDGF-A, TSP-1, and VEGFA 103. Additionally, H3K18 lactylation has been implicated in promoting arsenic-associated idiopathic pulmonary fibrosis progression via the YTHDF-1/N6-methyladenosine (m6A)/NREP pathway 115. In the pulmonary fibrotic microenvironment, increased glycolysis induced by airborne fine particulate matter (PM2.5) elevates lactylation levels at the promoters of pro-fibrotic genes, such as TGF-β, VEGFA, and PDGF-A, in macrophages. Notably, inhibition of lactylation using LDHA inhibitors has been shown to ameliorate PM2.5-induced pulmonary fibrosis 116. Consistently, Liu *et al*. reported that suppressing lactylation alleviates the progression of pulmonary fibrosis 117, a finding that aligns with recent comprehensive analyses identifying lactylation as a conserved driver of pathogenesis across broad respiratory diseases 118. These findings suggest that targeting lactylation to mitigate pulmonary fibrosis could represent a potential therapeutic approach for preventing the progression of pulmonary hypertension.

### Lactate/Lactylation and Vasoconstriction in PH

Abnormal vasoconstriction represents an early event in the pathogenesis of PH 119. Excessive vasoconstriction raises pulmonary resistance and pressure, working in tandem with vascular remodeling to advance PH from functional impairment to irreversible damage. Endothelial cells are key regulators of vascular tone. An imbalance in vasoactive factor secretion is fundamental to PH pathogenesis. Endothelin-1 (ET-1), a potent vasoconstrictor, is overexpressed in PH patients.

GPR81, a lactate receptor, is implicated in this process. Elevated lactate levels activate GPR81 on endothelial cells, prompting localized vesicular ET-1 release and contributing to vasoconstriction 120. Supporting this, Jones *et al*. showed that GPR81 activation boosts ET-1 synthesis in arterial smooth muscle cells. Secreted ET-1 binds endothelin-a receptors, causing vasoconstriction 121. Additionally, the direct infusion of exogenous sodium lactate in rats caused a rapid but transient increase in blood pressure, further linking lactate to vasoconstrictive effects 122. These findings suggest that lactate mediates vasoconstriction and may contributes to PH pathogenesis, highlighting its potential as a therapeutic target.

Finally, lactylation may play a critical role in neural impulse propagation 123. Although excessive sympathetic activation and parasympathetic suppression are well-recognized drivers of pulmonary hypertension 124, the metabolic underpinnings remain unclear. We postulate that aberrant lactylation within the autonomic nervous system could be a potential upstream mechanism contributing to this neurohormonal imbalance. Therefore, lactylation may regulate vascular tone by modulating nervous system control of vasodilation and vasoconstriction.

### Lactate/Lactylation and PASMCs Ferroptosis in PH

Ferroptosis, an iron-dependent form of regulated cell death characterized by the accumulation of lipid peroxides, has recently emerged as a critical mechanism in the pathogenesis of PH 125, 126. In this context, ferroptosis exerts a context-dependent “double-edged sword” effect 127: while targeted induction of ferroptosis in hyperproliferative PASMCs could theoretically reverse remodeling, uncontrolled ferroptosis leads to deleterious vascular injury 128, 129. Emerging evidence suggests that imbalanced ferroptosis triggers the release of damage-associated molecular patterns (DAMPs), thereby exacerbating perivascular inflammation. Simultaneously, under hypoxic or pathological stimuli, dysregulated non-coding RNAs act as key regulators by interfering with Glutathione Peroxidase (GPX4)-mediated antioxidant defense and acyl-CoA synthetase long-chain family member 4 (ACSL4)-mediated lipid metabolism 130, 131, leading to Fe^2+^ overload and the accumulation of lipid peroxidation products malondialdehyde (MDA). This cascade of metabolic dysregulation not only directly induces the phenotype switching of PASMCs from a contractile to a highly proliferative and migratory synthetic phenotype 132 but also causes significant medial wall thickening, ultimately driving PH disease progression. Thus, deciphering the metabolic triggers of ferroptosis is essential for understanding PH pathophysiology.

Lactate accumulation, resulting from the glycolytic shift, creates a metabolic microenvironment conducive to ferroptosis 133. The excessive production of lactate is often accompanied by an increase in mitochondrial reactive oxygen species (ROS) and altered iron metabolism 129, 133. Specifically, lactate accumulation contributes to an acidic microenvironment, which can promote the release of free iron from ferritin (ferritinophagy) or alter the function of transferrin receptors, thereby increasing the labile iron pool required for the Fenton reaction. Furthermore, the conversion of pyruvate to lactate by LDHA regenerates NAD^+^, which is essential for maintaining glycolytic flux. However, this shift away from mitochondrial metabolism may alter the NADPH/NADP^+^ ratio, which is critical for reducing glutathione disulfide (GSSG) to glutathione (GSH) 134. Since GPX4 relies on GSH to neutralize lipid peroxides, lactate-driven metabolic reprogramming may indirectly compromise GPX4 efficiency, rendering PASMCs more susceptible to ferroptotic stimuli.

Crucially, recent studies have unveiled that lactylation serves as a direct molecular switch linking metabolic stress to ferroptosis. Li *et al*. 72 reported that hypoxia-upregulated *ca-circSCN8A* recruits EP300 to promote FUS lactylation in PASMCs. This specific lactylation event induces the LLPS of FUS, which subsequently facilitates the formation of R-loops (DNA-RNA hybrids) causing genomic instability and crucially represses the transcription of SLC7A11. The repression of SLC7A11 limits cysteine uptake and GSH synthesis, thereby sensitizing cells to lipid peroxidation and ferroptosis-mediated vascular injury. This finding is significant as it delineates a novel “Hypoxia-Lactate-FUS Lactylation-Ferroptosis” axis, highlighting that lactylation regulates cell fate not only through gene transcription but also through phase separation and genomic stability. Furthermore, studies report that H3K18la upregulates the transcription of ACSL4 and LC3 in pulmonary microvascular endothelial cells, concurrently increasing PUFA substrates and the labile iron pool 135. Although this phenomenon was identified in sepsis-induced acute lung injury, given the crucial role of endothelial dysfunction in PH, this lactylation-dependent pro-ferroptotic mechanism offers a highly plausible clue for future PH research. In summary, the lactate-lactylation axis appears to function as a “metabolic switch” for ferroptosis in PH. While the EP300/FUS pathway provides the first solid molecular evidence, we hypothesize that broad protein lactylation likely remodels the ferroptosis landscape by modifying canonical regulators (e.g., GPX4, ACSL4). Clarifying these interactions will determine whether targeting lactylation promotes beneficial cell death in hyperproliferative PASMCs or prevents deleterious vascular injury.

### Lactate/Lactylation and Right Ventricular Remodeling

While pulmonary vascular remodeling initiates the pathogenesis of pulmonary hypertension (PH), the functional state of the right ventricle (RV) ultimately dictates patient prognosis. Similar to pulmonary vascular cells, the RV undergoes profound metabolic reprogramming under pressure overload, shifting from fatty acid oxidation to glycolysis to maintain contractility 136. However, this adaptive mechanism eventually becomes maladaptive, leading to robust lactate accumulation. Metabolomic analyses have revealed significantly elevated lactate levels in the RV tissues of PH animal models, which directly correlate with the degree of RV hypertrophy and dysfunction 58. Clinically, plasma lactate has been identified as a critical metabolic hallmark specifically associated with RV failure in PH patients, serving as a stronger predictor of mortality and poor prognosis than traditional hemodynamic parameters alone 52, 56.

Persistent and profound lactate overload in the failing RV provides substrates for protein lactylation. Although direct investigations into specific lactylated targets within the PH-RV remain in their infancy, recent extensive cardiovascular studies offer compelling mechanistic insights 137, 138. Aberrant lactylation has been demonstrated to fundamentally drive maladaptive cardiac remodeling; for instance, H3K18la-mediated modification upregulates pro-hypertrophic genes (e.g., ANP, BNP, and β-MHC) 139, while Snail1 lactylation activates TGF-β/Smad2 signaling to promote aggressive interstitial fibrosis 140. Given that cardiomyocyte hypertrophy and interstitial fibrosis are the core pathological features of RV decompensation in PH, it is highly plausible that the dysregulated lactate-lactylation axis acts as a critical, yet underexplored, pathological driver in this progression. Importantly, this metabolic derangement is not merely a byproduct of RV failure, but a reversible pathological mechanism. Experimental evidence demonstrates that preventing pathological lactate accumulation—for instance, via the pyruvate dehydrogenase kinase inhibitor Dichloroacetate (DCA)—not only regresses pulmonary vascular remodeling but also directly ameliorates RV function by restoring metabolic flexibility and homeostasis 141. Consequently, unraveling the precise lactylation networks within the RV may yield novel prognostic biomarkers and elucidate the molecular basis for restoring cardiac resilience, thereby laying a crucial mechanistic foundation for the development of future targeted interventions.

### Targeted Therapeutic Strategies

Given the role of lactate and lactylation in driving pulmonary vascular remodeling, targeting this metabolic-epigenetic pathway offers a promising therapeutic strategy. Current pharmacological interventions primarily focus on three key dimensions: lactate metabolic enzymes, lactate transporters, and lactylation regulators.

### Lactate Metabolic Enzymes

Regulating the enzymes responsible for lactate production and clearance is a direct therapeutic approach.

#### Inhibition of Glycolysis and LDHA

Blocking the source of lactate generation has shown potent anti-remodeling effects. Hexokinase 2 (HK2), the first rate-limiting enzyme of glycolysis, was recently identified as a critical target; its specific knockdown significantly attenuated endothelial-mesenchymal transition (EndMT) and vascular remodeling by abolishing lactylation^142^. Similarly, targeting the upstream regulator CTRP1 to reactivate AMPK signaling has been proven to suppress glycolytic flux and lactate production, thereby inhibiting PASMC hyperproliferation 80. Furthermore, direct inhibition of LDHA with small molecules like oxamate effectively reduces the intracellular lactate pool. This blockade suppresses the “Warburg effect” and prevents the specific lactylation events that drive the expression of proliferative genes such as *Pgf* and *Ythdf2* 70.

#### Activation of PDH

Conversely, promoting the oxidative metabolism of pyruvate reduces lactate accumulation. Dichloroacetate (DCA), a well-known PDK inhibitor, activates PDHA1 to restore mitochondrial respiration. In PH, DCA has been proven to reverse metabolic reprogramming, decrease lactate levels, and improve right ventricular function 74.

### Lactate Transport

MCTs mediate the critical transmembrane flux of lactate; thus, inhibiting these transporters effectively disrupts intracellular and extracellular lactate homeostasis. Although clinical applications are still evolving, the therapeutic logic is compelling: blocking MCTs prevents the uptake of exogenous lactate by pulmonary vascular cells. This blockade depletes the intracellular pool of lactate available for conversion into lactyl-CoA, thereby "starving" the epigenetic machinery required for lactylation and mitigating the aberrant proliferative response driven by metabolic stress 44, 67

### Lactylation regulators

Directly targeting the “writer” enzymes responsible for adding lactyl groups to proteins represents a precise epigenetic intervention strategy. The acetyltransferase EP300 serves as a primary lactylation writer, and its inhibition offers a more specific approach than broad metabolic blockade.

#### EP300 Inhibition

Pharmacological inhibitors such as C646 have demonstrated significant efficacy in recent breakthrough studies. Li *et al*. revealed that C646 competitively binds to the catalytic site of EP300, effectively blocking the lactylation of the RNA-binding protein FUS. This intervention disrupts the formation of pathological LLPS condensates, restores the expression of the antioxidant gene SLC7A11, and ultimately ameliorates ferroptosis-driven pulmonary vascular remodeling 72.

## Conclusion and Future Perspectives

In conclusion, PH is a multifactorial disease where metabolic reprogramming is increasingly recognized as a key pathophysiological feature. We suggest that the “lactate-lactylation process” may serve as an important bridge connecting glycolytic dysregulation to the five pathological pillars of PH: aberrant PASMC proliferation, immune-inflammatory dysregulation, pulmonary fibrosis, abnormal vasoconstriction and PASMC Ferroptosis. Importantly, this metabolic derangement extends to the right ventricle, driving the cardiac transition from adaptive to maladaptive remodeling. Furthermore, preclinical studies indicate that pharmacological interventions targeting this pathway—such as inhibiting lactate production, blocking lactate transport, or modulating lactylation writers—hold promise for simultaneously mitigating pulmonary vascular remodeling and improving right ventricular function, particularly in PAH and hypoxic PH.

However, translating these findings into clinical practice warrants careful consideration. First, given that lactate can serve as an essential energy substrate for the heart and brain, systemic inhibition of lactate metabolism might pose risks to PH patients with compromised hemodynamics. Second, since lactylation regulators such as EP300 also govern histone acetylation, the selectivity of targeting lactylation remains a challenge that needs to be addressed to avoid potential off-target effects. Therefore, future investigations would benefit from focusing on lung-targeted drug delivery systems and elucidating the precise downstream targets of lactylation.

## Figures and Tables

**Table 1:** Lactate and Lactylation Changes in Pulmonary Hypertension

	Model/Subtype	Lactate/ Lactylation Changes	References
**PH Patients**	PH associated with COPD	Arterial blood lactate levels show a positive correlation with pulmonary arterial systolic pressure.	55
	PH with right heart failure	Plasma lactate is considered a hallmark feature of right ventricular failure.	52
	PH (General)	Serum lactate levels are significantly elevated; hyperlactatemia is an independent predictor of mortality.	52-54, 56
**Animal Models**	Hypoxia-induced PH (Mice)	Elevated lactate levels in lung and right ventricular tissues.	58
	Hypoxia combined with SU5416-induced PH (Mice)	Elevated lactate levels in lung and right ventricular tissues.	58
	Monocrotaline-induced PH (Rats)	Inhibition of LDHA can mitigate vascular remodeling and right ventricular dysfunction.	59, 60
	Hypoxia-induced PH (Rats)	Increased lactylation of histones H3K18 and H4K5 in lung tissue. Increased L-lactylation	67 81
**Cell Models**	PASMCs and PAECs isolated from PH patients	Increased ¹³C-labeled lactate production.	61
	Hypoxia-stimulated HPASMCs	FUS Lactylation Increased H3K18 Lactylation Increased (IL-1β etc. promoter region)	72 105
	HPASMCs	Lactate accumulation induced by hypoxia triggers histone lactylation at the PDE4B promoter, promoting vascular remodeling.	71
	Hypoxia-stimulated PASMCs (Rats)	Increased lactate production. Increased lactylation of pan-Kla and H3K18 Increased H4K5la.	67 67,70 70

Abbreviations used in the table: COPD:chronic obstructive pulmonary disease; PH:pulmonary hypertension; PASMCs:pulmonary artery smooth muscle cells; PAECs:pulmonary artery endothelial cells; HPASMCs:human pulmonary artery smooth muscle cells.
